# Development of 3D printable graphene oxide based bio-ink for cell support and tissue engineering

**DOI:** 10.3389/fbioe.2022.994776

**Published:** 2022-10-18

**Authors:** Jianfeng Li, Xiao Liu, Jeremy M. Crook, Gordon G. Wallace

**Affiliations:** ^1^ ARC Centre of Excellence for Electromaterials Science, Intelligent Polymer Research Institute, University of Wollongong, Wollongong, NSW, Australia; ^2^ Max Planck Institute of Microstructure Physics, Halle, Germany; ^3^ Max Planck-University of Toronto Centre for Neural Science and Technology, Toronto, ON, Canada; ^4^ Illawarra Health and Medical Research Institute, University of Wollongong, Wollongong, NSW, Australia; ^5^ Arto Hardy Family Biomedical Innovation Hub, Chris O'Brien Lifehouse, Camperdown, NSW, Australia; ^6^ School of Medical Sciences, Faculty of Medicine and Health, The University of Sydney, Camperdown, NSW, Australia

**Keywords:** 3D bioprinting, bioink, biomaterials, stem cells, graphene, tissue engineering

## Abstract

Tissue engineered constructs can serve as *in vitro* models for research and replacement of diseased or damaged tissue. As an emerging technology, 3D bioprinting enables tissue engineering through the ability to arrange biomaterials and cells in pre-ordered structures. Hydrogels, such as alginate (Alg), can be formulated as inks for 3D bioprinting. However, Alg has limited cell affinity and lacks the functional groups needed to promote cell growth. In contrast, graphene oxide (GO) can support numerous cell types and has been purported for use in regeneration of bone, neural and cardiac tissues. Here, GO was incorporated with 2% (w/w) Alg and 3% (w/w) gelatin (Gel) to improve 3D printability for extrusion-based 3D bioprinting at room temperature (RT; 25°C) and provide a 3D cellular support platform. GO was more uniformly distributed in the ink with our developed method over a wide concentration range (0.05%–0.5%, w/w) compared to previously reported GO containing bioink. Cell support was confirmed using adipose tissue derived stem cells (ADSCs) either seeded onto 3D printed GO scaffolds or encapsulated within the GO containing ink before direct 3D printing. Added GO was shown to improve cell-affinity of bioinert biomaterials by providing more bioactive moieties on the scaffold surface. 3D cell-laden or cell-seeded constructs showed improved cell viability compared to pristine (without GO) bio-ink-based scaffolds. Our findings support the application of GO for novel bio-ink formulation, with the potential to incorporate other natural and synthetic materials such as chitosan and cellulose for advanced *in situ* biosensing, drug-loading and release, and with the potential for electrical stimulation of cells to further augment cell function.

## 1 Introduction

Human tissue engineering offers new opportunities in research and medicine to model, restore, maintain, or improve tissues of the body. The amalgamation of mammalian cells with biomaterials is especially enabling, especially when combined with 3D bioprinting (Moysidou et al., 2021). Self-renewing and multi-lineage stem cells are particularly amenable to tissue engineering (Polak and Bishop, 2006; Yan et al., 2020), although there can be technical and ethical challenges depending on the type of stem cell used (Kuo and Wang, 2013; Sun et al., 2014). Adipose tissue derived stem cells (ADSCs) are considered less problematic, because they are easier to obtain with minimally invasive techniques and able to be used for both autologous and allogeneic therapies (Frese et al., 2016).

As a polysaccharide derived from brown seaweed, sodium alginate (Alg) has tuneable rheological properties that can be further manipulated through ionic cross-linking. This makes Alg an excellent candidate for 3D bioprinting (Lee and Mooney, 2012; Axpe and Oyen, 2016). However, mammalian cells, such as human ADSCs, cannot adhere to the surface of pristine Alg, limiting its role as a cell support material for tissue engineering (Andersen et al., 2015). On the other hand, graphene and its derivatives have gained much interest in materials research over the past decade due to their excellent physicochemical properties (Dideikin and Vul', 2019; Passaretti, 2022). For bionic applications, graphene has been shown to have outstanding biocompatibility, with the added potential to act as a depot for drug delivery and/or electrical stimulation (Thompson et al., 2015; Kadumudi et al., 2021). However, widely investigated graphene substrates for biomedical research are in a two-dimensional (2D) format, limiting their performance (Magne et al., 2021). 3D printing is a promising technology for 3D graphene scaffold fabrication and tissue engineering. However, graphene has generally been utilized as an additive for ink preparation without cells (Wu et al., 2021), and it has not been thoroughly studied as an additive for cell laden bioink preparation and 3D printing.

Here, we report the combination of 3D printable graphene oxide (GO) using Alg and gelatin (Gel) as the basis of a novel bio-ink to support human ADSCs. Gel was incorporated in the bioink to increase the 3D printability of Alg based composites and could be removed after long term incubation in the culture medium (CM) or at high temperature to dissolve in water after 3D printing (Kyle et al., 2017; Li et al., 2020). Generally, chemical modification is required for the bioactivity improvement of Alg and structure stability of Gel, which may involve complicated chemical reactions and tedious purification processes (Bao et al., 2020; Neves et al., 2020). We have optimised the ink through systematic investigation into the effects of varying the GO concentration on cell affinity and viability. Although GO has recently been incorporated with cell laden Alg for bone tissue engineering, previously reported inks require either pre-crosslinking or an additional additive (glycerol) for ink preparation (Choe et al., 2019; Zhang et al., 2021). With the GO/Alg/Gel/glycerol/phosphate buffered saline ink, GO was not uniformly dispersed within the ink, with GO aggregates visible by optical microscopy (Zhang et al., 2021). Furthermore, in both cases, the GO containing inks have not been tested with widely used cell seeding methods for 3D cell culture (Choe et al., 2019; Zhang et al., 2021). On the contrary, our synthesized GO was uniformly distributed in the Alg/Gel solution over a wide concentration range (0.05%–0.5%, w/w) and the GO containing ink supported 3D printing of ADSCs and seeding of cells after printing, with cells remaining viable and proliferative for ensuing culture and maintenance of constructs.

## 2 Materials and methods

### 2.1 Graphene oxide preparation

GO was synthesized from graphite powder with a modified Hummers method as reported in our previous papers (Li et al., 2019; Li et al., 2020). Briefly, 38 ml 98% sulfuric acid (H_2_SO_4_; Chem-Supply, Australia) was mixed with 0.5 g graphite powder (325 mesh, 99.95%; Aladdin Ltd., China) in an ice bath. 0.25 g sodium nitrate (NaNO_3_; Sigma-Aldrich, United States) and 1.25 g potassium permanganate (KMnO_4_; Chem-Supply, Australia) were then added gradually with vigorous agitation. The reaction mixture was continuously stirred for 5 days at RT, followed by addition of 75 ml diluted 5% H_2_SO_4_, and heated to 90°C for 2 h. After the reaction, the mixture was cooled down to RT and the reaction was terminated with the addition of 30% hydrogen peroxide (H_2_O_2_; Chem-Supply, Australia) and washed with 1 M hydrochloric acid (HCl; Chem-Supply, Australia) 10 times. Additional purification was carried out by 1-week dialysis with a dialysis membrane (MWCO = 14,000 Da; Sigma-Aldrich, United States), and 0.45% (w/w) GO aqueous dispersion was obtained with subsequent exfoliation by an ultrasonicator (Unisonics cleaner, Australia). 18 MΩ Milli-Q water (MQ-H_2_O) was used to prepare all experimental solutions.

### 2.2 Adipose tissue derived stem cell culture

ADSCs were purchased from Lonza (Australia) and cultured in cell CM consisting of Gibco Dulbecco’s Modified Eagle Medium (DMEM; Thermo Fisher, Australia) supplemented with 10% fetal bovine serum (FBS; Thermo Fisher, Australia), 1 ng/ml basic fibroblast growth factor (bFGF; Thermo Fisher, Australia), 1% penicillin-streptomycin (P/S; Thermo Fisher, Australia), 1% non-essential amino acids (NEAA; Thermo Fisher, Australia) in a cell culture incubator (37°C with humidified 5% CO_2_) as described previously (Li et al., 2017).

### 2.3 Alginate/gelatin/graphene oxide ink preparation and 3D printing

For 10.000 g 2%/3%/0.3% (w/w) Alg/Gel/GO ink preparation, 200 mg sodium alginate (Alg; M_W_: 80,000–120,000 Da, M/G = 1.56; viscosity of 2% w/w solution ≥2,000 cP at 25°C; Sigma-Aldrich, United States) and 6.667 g 0.45% (w/w) GO dispersion were mixed uniformly with additional 2.830 g MQ-H_2_O at 80°C for 2 h. Then, 300 mg gelatin (Gel; Sourced from bovine skin, Sigma-Aldrich, United States) was added and mixed for 1 h at 80°C. The as-prepared 10.000 g 2%/3%/0.3% (w/w) Alg/Gel/GO ink was subsequently loaded into a syringe barrel (Nordson EFD, United States) for 3D printing and centrifuged at 300 x g for 2 min (Thermoline K241 centrifuge, Australia) to remove air bubbles from the ink. The ink could be stored at 4°C for more than 1 month. Bio-inks with different GO ratios (0.05%–0.3%, w/w) were prepared with specific amounts of each component, accordingly, as shown in Table 1. The highest GO concentration for Alg/Gel/GO ink achieved was 0.5% (w/w), with a higher GO concentration resulting in undispersed GO content in the ink that may induce nozzle clogging during printing. GO dispersion of 10.000 g 2%/3%/0.5% (w/w) Alg/Gel/GO ink was prepared by concentrating 11.111 g 0.45% (w/w) GO MQ-H_2_O dispersion to 9.500 g through solvent evaporation at 80°C or directly dispersing 50 mg freeze-dried GO in 9.450 g MQ-H_2_O with subsequent heating and ultrasonication.

**TABLE 1:** Formulation of Alg/Gel/GO ink with different GO concentrations.

Ingredient	Weight of each ingredient			
	2%/3%/0.05%(w/w)Alg/Gel/GO	2%/3%/0.1%(w/w)Alg/Gel/GO	2%/3%/0.2%(w/w)Alg/Gel/GO	2%/3%/0.3%(w/w)Alg/Gel/GO
Alg	**200 mg**	**200 mg**	**200 mg**	**200 mg**
Gel	**300 mg**	**300 mg**	**300 mg**	**300 mg**
0.45% (w/w) GO	**1.111 g**	**2.222 g**	**4.444 g**	**6.667 g**
Water	**8.389 g**	**7.278 g**	**5.056 g**	**2.833 g**

Printing was performed using a 3D Bioplotter (EnvisionTEC GmbH, Germany). The syringe barrel with loaded Alg/Gel/GO ink was kept in the printing magazine of the printer at RT prior to printing layer-by-layer at RT. The syringe barrel was fitted with a stainless-steel nozzle (Inner diameter: 200 μm; Nordson EFD, United States) for printing of a cubic model (10 mm × 10 mm × 1 mm) onto a petri dish fixed on a cold stage (5°C). The air pressure applied for extrusion is 5.0 bar with a feeding speed of printing at 15 mm/s. 3D printed scaffolds were immersed in 2% (w/w) calcium chloride (CaCl_2_; Chem-Supply, Australia) solution for crosslinking (10 min) and the Gel component was removed in hot MQ-H_2_O (80°C, 3 h) thereafter only for ADSC seeding experiment (Section 3.3).

### 2.4 Rheology

Samples were prepared 1 day before rheological testing and characterized on an AR-G2 rheometer (TA Instruments, United States) at RT with a 2°/40 mm steel cone and plate geometry. Storage modulus (G′) and loss modulus (G″) of samples were measured within a frequency range of 0.01–10 Hz at RT.

### 2.5 Scanning electron microscopy

3D printed scaffolds were frozen in liquid nitrogen for 35 s prior to microstructure imaging with a JEOL JSM-6490LV scanning electron microscope (SEM). GO-containing 3D printed scaffolds were immersed in 5 mM ascorbic acid (BDH Chemicals, Australia) solution for 3 h at 80°C to remove Gel component and increase electrical conductivity from chemical reduction of GO to improve imaging quality. Cell laden inks/scaffolds were fixed in 3.7% paraformaldehyde (PFA) solution for 10 min in advance and subsequently observed under SEM as described.

### 2.6 Adipose tissue derived stem cell seeding and culture on 3D printed scaffolds

3D printed scaffolds were sterilized by immersing in 70% (v/v) ethanol solution for 2 h and then dried in a biosafety cabinet (BSC) with subsequent sterilization under UV for 1 h before use. The sterilized scaffolds were immersed in CM for 1 day before ADSC seeding (seeding density: 5 × 10^5^ cells/mL). CM was refreshed every 2 days during culture.

### 2.7 3D printing adipose tissue derived stem cell-laden bio-inks

**FIGURE 1:**
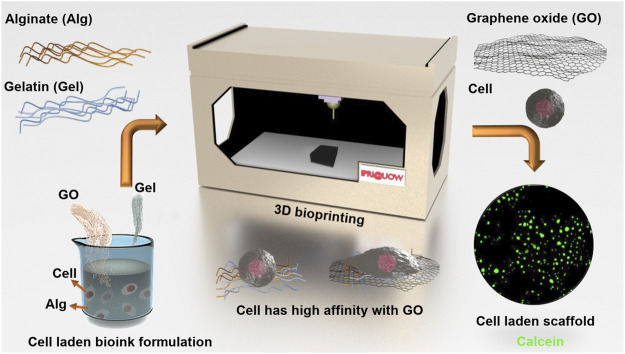
Schematic of ADSC-laden Alg/Gel/GO bio-ink preparation and 3D bioprinting.

Under sterilized conditions, the Alg/Gel/GO ink could be used for living cell printing directly (Figure 1). In detail, freeze-dried GO was immersed in 70% (v/v) ethanol solution for 2 h, followed by drying in a biosafety cabinet (BSC) and subsequent sterilization under UV for 1 h. Alg and Gel powders were sterilized in a BSC under UV for 1 h. For 5.000 g 2%/3%/0.05% (w/w) Alg/Gel/GO ink preparation, 100 mg Alg was dissolved in 4.735 g DMEM at 80°C with magnetic agitation for 3 h, and thereafter 2.5 mg GO was dispersed in the solution with ultrasonication. After both Alg and GO were completely dissolved and dispersed, 150 mg Gel was added and dissolved with magnetic agitation at 80°C. The as-prepared 2%/3%/0.05% (w/w) Alg/Gel/GO DMEM ink could be stored for at least 1 month at 4°C and should be pre-warmed to 37°C before mixing with ADSCs at a density of 2 × 10^6^ cells/ml. The ADSC laden Alg/Gel/GO ink was loaded into a syringe barrel and centrifuged at 300 x g for 2 min to remove air bubbles in the ink. The printing of ADSC-laden Alg/Gel/GO ink was similar to the printing of Alg/Gel/GO ink, while the air pressure applied was reduced to 4.0 bar. ADSCs laden pristine Alg/Gel scaffolds were printed with the same procedures, but at a pressure of 4.3 bar 3D printed ADSC-laden scaffolds were crosslinked in 2% (w/w) CaCl_2_ solution for 1 min before being transferred into CM for long term culture in an incubator (37°C with humidified 5% CO_2_). CM was refreshed every 2 days during culture.

For cell viability and morphology testing in the ink, 200 μl ADSC-laden (2 × 10^6^ cells/ml) 2%/3% (w/w) Alg/Gel and 2%/3%/0.05% (w/w) Alg/Gel/GO ink were deposited into wells of a 24-well plate (Corning, United States) and crosslinked with 2% (w/w) CaCl_2_ solution for 1 min before replacing the crosslinking bath with fresh CM.

### 2.8 Evaluation of adipose tissue derived stem cell viability

Calcein AM (5 μg/ml; Thermo Fisher, Australia) and propidium iodide (PI, 1 μg/ml; Thermo Fisher, Australia) were used for evaluation of ADSC viability as per the manufacturer’s manual. Samples were incubated with the assay for 30 min in an incubator (37°C with humidified 5% CO_2_) and washed with fresh CM before imaging. AxioImager microscope (Zeiss, Germany) was used for imaging and analysis.

### 2.9 Proliferation analysis of adipose tissue derived stem cells in scaffolds of 3D printed cell-laden bio-ink

PrestoBlue cell viability reagent (Thermo Fisher, Australia) was used to assess the proliferation of 3D printed ADSCs (ie. of scaffolds produced from cell-laden bio-inks) at different time points as per the manufacturer`s instructions. For each measurement of each type of scaffold, three 3D printed ADSC-laden scaffolds were incubated with the reagent for 30 min in an incubator and the supernatant was transferred into a 96-well plate for subsequent fluorescence intensity measurement with a microplate reader (ex/em: 544/590; POLARstar Omega, Germany). After 5 days, the 3D printed ADSC-laden scaffold was fragile to handle and not possible for proliferation analysis thereafter.

### 2.10 Statistical analysis

OriginPro 2019 was used for data graphing and statistical analysis. Unless specified, all the quantitative data are presented as mean ± standard deviation (SD). Brown-Forsythe test was employed to verify the variance homogeneity of data to confirm the statistical assumptions for ANOVA were satisfied. If the assumption was met (*p* > 0.05), a significance level of 0.05 was used for two-way ANOVA (Bonferroni *post hoc* test). If not, a significance level of 0.01 was used for higher stringency.

## 3 Results and discussion

### 3.1 Rheological properties of the alginate/gelatin/graphene oxide ink

**FIGURE 2:**
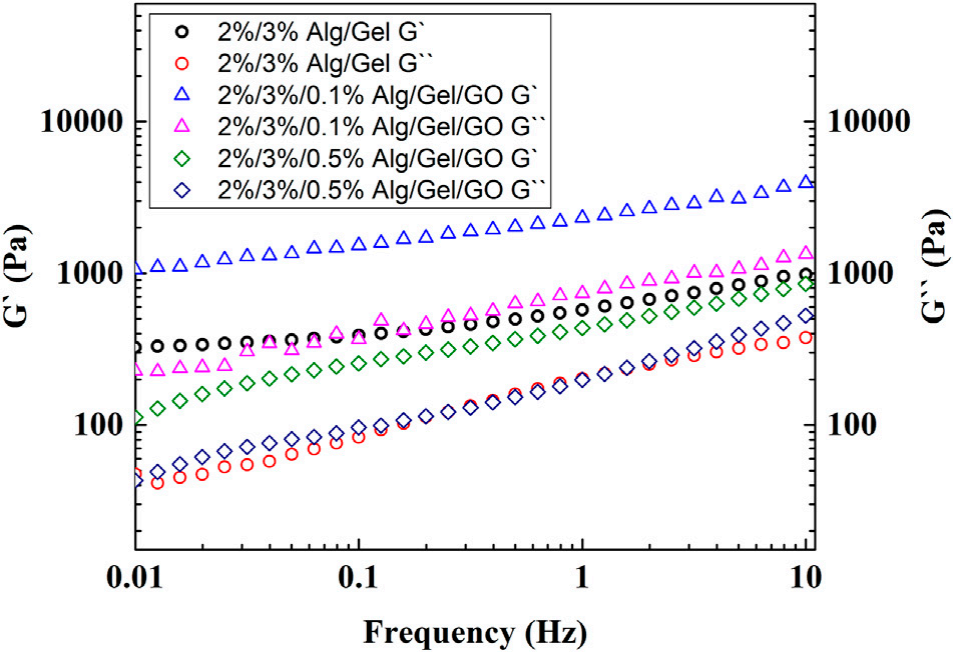
Rheological measurements of 2%/3% (w/w) Alg/Gel, 2%/3%/0.1% (w/w) Alg/Gel/GO, and 2%/3%/0.5% (w/w) Alg/Gel/GO with frequency sweep from 0.01 to 10 Hz.

The ink was prepared as described in the Experimental Section and the rheological properties were investigated. These properties serve as important criteria for 3D printable ink design. Specifically, storage modulus (G`) refers to the elastic properties, serving as a measure of elastic shape retention, while loss modulus (G``) refers to the viscous properties, serving as a measure of flowability (Schwab et al., 2020). 2%/3%/0.1% (w/w) Alg/Gel/GO and 2%/3%/0.5% (w/w) Alg/Gel/GO inks were used for rheological study in comparison to the pristine 2%/3% (w/w) Alg/Gel ink. 2%/3%/0.5% (w/w) Alg/Gel/GO was chosen because it`s the ink with the highest GO ratio introduced in the paper, and 2%/3%/0.1% (w/w) Alg/Gel/GO was chosen because it has an intermediate GO ratio compared to the other two inks. Both Alg and Gel are viscoelastic materials, and Alg/Gel solutions (2%/3%; w/w) showed higher storage modulus (G`) than loss modulus (G``) over the frequency range tested (Figure 2), indicating it could retain structure after being extruded from the nozzle (Li et al., 2020).

With the addition of GO [0.1% or 0.5% (w/w)], the dispersion showed higher storage modulus (G`) than loss modulus (G``), indicating that the addition of GO up to 0.5% (w/w) does not influence printability. Storage modulus increased with addition of 0.1% (w/w) GO to the 2%/3% (w/w) Alg/Gel solution but decreased with addition of 0.5% (w/w) GO. This suggests that a lower GO content serves to interconnect Alg and Gel polymer chains through hydrogen bonding with an increased capacity to store elastic energy, while higher GO content inhibits the internal hydrogen bonding between polymers, decreasing elastic energy storage with negligible influence on the viscous behaviour of the ink (G``) (Chen et al., 2018).

### 3.2 Morphology of 3D printed scaffolds without cells

**FIGURE 3:**
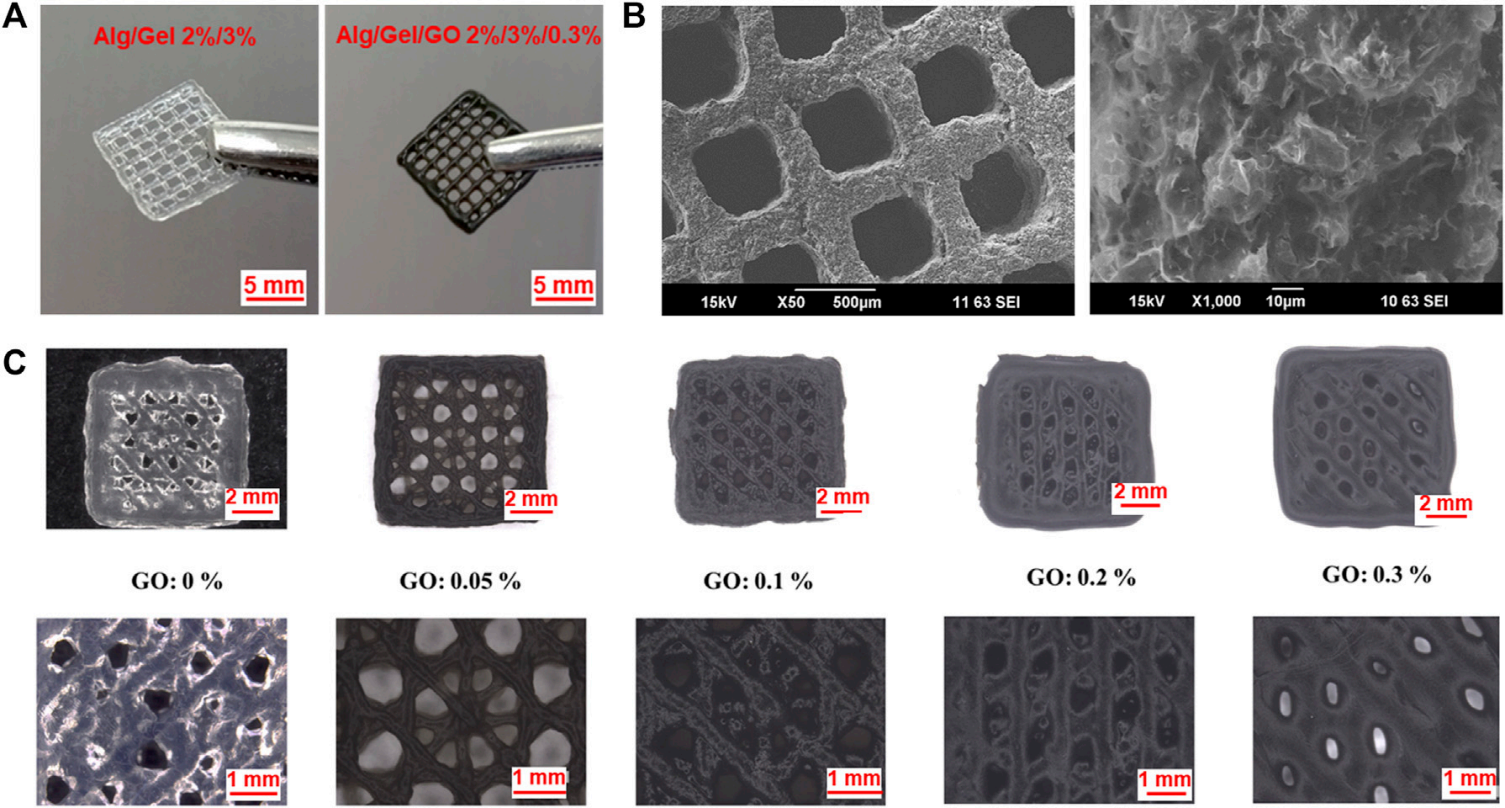
Morphology of 3D printed Alg/Gel/GO scaffolds. Photographs of structures obtained using the following bioink compositions **(A)** Alg/Gel (2%/3%, w/w) and Alg/Gel/GO (2%/3%/0.3%, w/w). **(B)** SEM images of 3D printed structures obtained using 2%/3%/0.3% (w/w) Alg/Gel/GO at different magnifications. **(C)** Optical images of 3D printed scaffolds with different GO ratios printed under the same conditions.

Both 2%/3% (w/w) Alg/Gel ink solution and 2%/3%/0.3% (w/w) Alg/Gel/GO ink dispersion gelled after cooling from 80°C to RT (Supplementary Figures S1A,B). 3D Alg/Gel scaffolds and 3D Alg/Gel/GO scaffolds were 3D printed according to the procedure described in the Experimental Section (Supplementary Figure S2A). Both scaffolds were mechanically robust after crosslinking, exhibiting a well-defined architecture (Figures 3A,B). However, 3D printed Alg based scaffolds with cruciform pattern are prone to collapsing with reduced capability for nutrition and waste transportation (Li et al., 2020).

So, scaffolds obtained using the 2%/3% (w/w) Alg/Gel ink with different GO concentrations were printed with multi-angle design as previously described (Figure 3C) (Li et al., 2020). The 3D printed scaffolds comprised a pore size ranging from ∼60 to ∼1,000 μm, with the potential to be tailored for various tissue engineering applications (Zaeri et al., 2022). Pristine (without GO) 2%/3% (w/w) Alg/Gel solutions can be 3D printed, while the scaffolds obtained using the 0.05% (w/w) and 0.1% (w/w) GO content inks exhibited improved resolution (Figure 3C) due to improved storage modulus to retain strut structure after extrusion and flowability during extrusion.

When the GO content exceeds 0.2%, the morphology deteriorates with decreased storage modulus, and GO flakes in the ink may hinder the crosslinking of Alg after printing as well. Applying the same 3D printing parameters, the strand diameter (522 μm) of 3D printed scaffolds using 2%/3%/0.3% (w/w) Alg/Gel/GO is 125% greater than scaffolds printed using 2%/3%/0.05% (w/w) Alg/Gel/GO (232 μm) (Supplementary Figure S2B). However, with well-tuned parameters, 2%/3%/0.3% (w/w) Alg/Gel/GO could be 3D printed with fine morphology (smooth surface and printed structure as designed) (Figure 3A). Also notable, microscopy analysis revealed increased loading of the ink with GO correlated with increased visibility of graphene flakes and material porosity (Supplementary Figures S3A–D).

### 3.3 Attachment and viability of adipose tissue derived stem cells seeded on 3D printed scaffolds

**FIGURE 4:**
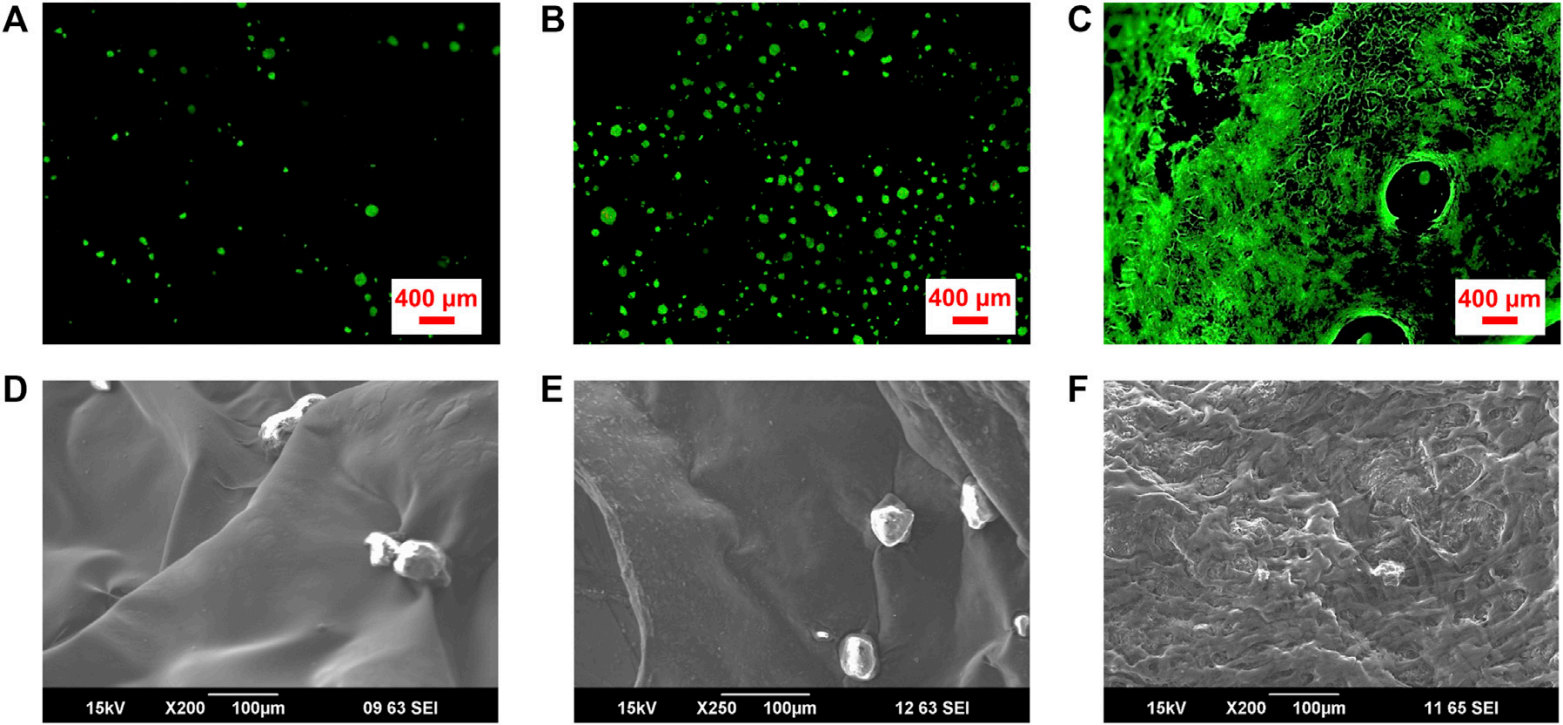
Attachment and survival of ADSCs seeded on 3D printed Alg/Gel scaffolds comprising different GO concentrations. Fluorescence microscope images of live (Calcein AM; green), and dead (PI; red) ADSCs cultured on porous 3D printed scaffolds with final compositions of **(A)** 2% (w/w) Alg, **(B)** 2%/0.05% (w/w) Alg/GO, and **(C)** 2%/0.3% (w/w) Alg/GO following 1-day culture, respectively. **(D–F)** SEM images of ADSCs on 3D printed scaffolds with final compositions of 2% (w/w) Alg, 2%/0.05% (w/w) Alg/GO and 2%/0.3% (w/w) Alg/GO following 1-day culture, respectively.

Cell seeding of 3D printed scaffolds following printing is a useful approach to tissue engineering, and it plays a pivotal role in cell colonization and the subsequent 3D tissue formation (Li et al., 2017; Liu et al., 2020). 3D scaffolds with high affinity for cells can elicit effective cell attachment after cell seeding, ensuring uniform cell distribution throughout the structure (Cámara-Torres et al., 2021). As shown in Figures 4A,B, coverage of a scaffold surface by seeded ADSCs increases 5.1 fold (*n* = 3), with increasing GO concentration from 0% to 0.05% (w/w). The scaffold with 0.3% (w/w) GO was for the most part covered with adherent cells (Figure 4C), comparable to the previously reported graphene coated Alg scaffolds (Li et al., 2020) and indicative of extensive exposure of GO moieties on the scaffold surface. Cells showed rounded morphology on the scaffolds with compositions of pristine 2% (w/w) Alg and 2%/0.05% (w/w) Alg/GO, while cells became flat on the scaffolds with the composition of 2%/0.3% (w/w) Alg/GO (Figures 4D–F). With 0.3% (w/w) GO content, cells displayed increased attachment and an extended morphology, indicating scaffolds with higher GO concentration promote extension and spreading of lamellipodia.

### 3.4 Adipose tissue derived stem cell laden ink development

**FIGURE 5:**
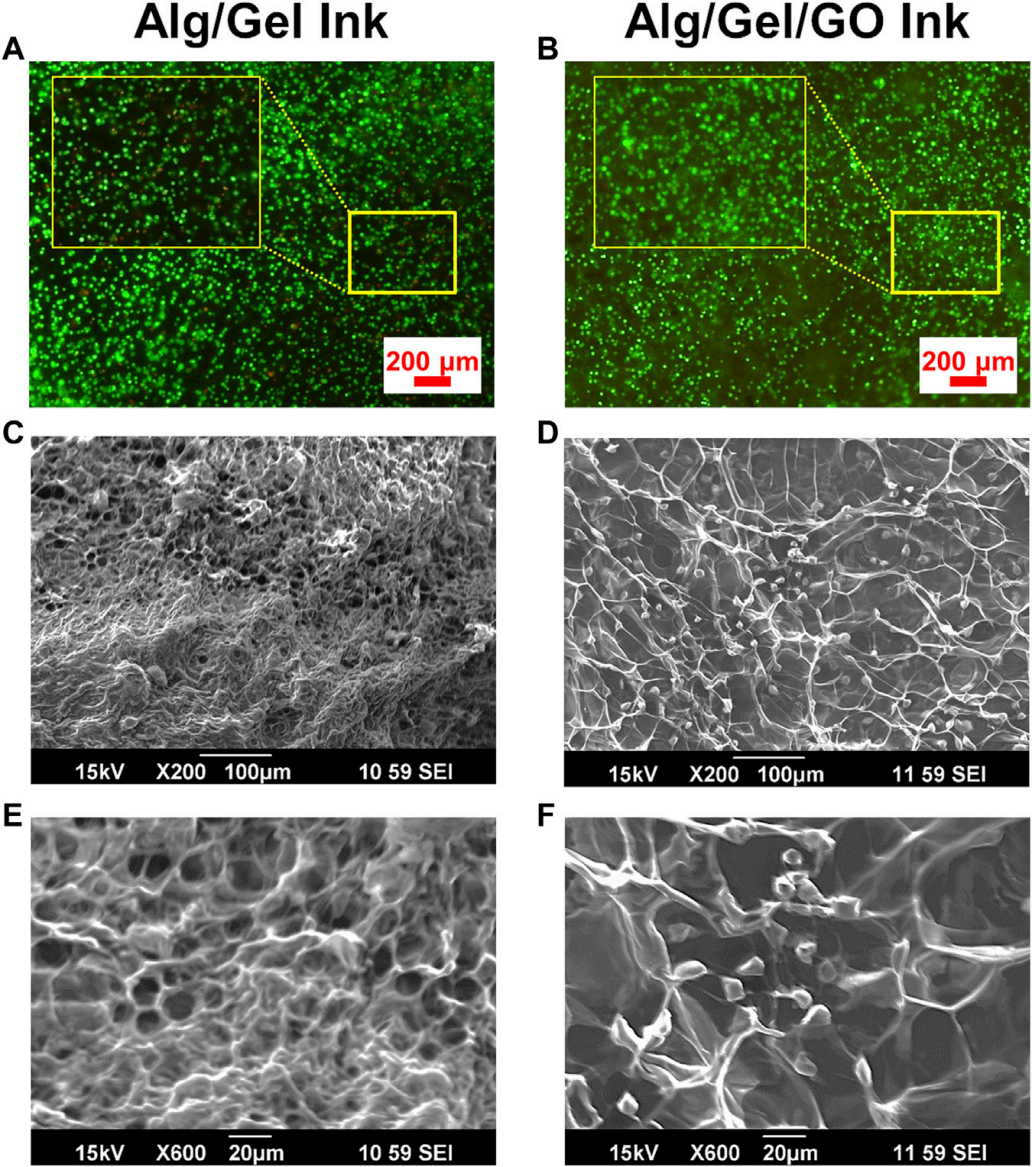
Improved ADSC viability in GO-containing inks. Fluorescence images of live (Calcein AM; green) and dead (PI; red) cell staining for ADSC-laden **(A)** pristine 2%/3% (w/w) Alg/Gel and **(B)** 2%/3%/0.05% (w/w) Alg/Gel/GO inks following culture for 1 day. SEM images of ADSC-laden **(C,E)** pristine 2%/3% (w/w) Alg/Gel and **(D,F)** 2%/3%/0.05% (w/w) Alg/Gel/GO inks at different magnifications following culture for 1 day.

GO has been shown to be beneficial for retaining and improving cell viability in tissue engineering (Savchenko et al., 2021). To further investigate the effect of GO on cell support for 3D bioprinting, ADSC-laden bio-ink was prepared with 2%/3%/0.05% (w/w) Alg/Gel/GO composite. Re-dispersing freeze-dried GO in aqueous solution is challenging and time consuming, so 0.05% GO was utilized in the experiment to evaluate the effect of GO addition on bioink preparation and performance. The presence of GO increased cell viability compared to pristine 2%/3% (w/w) Alg/Gel (Figures 5A,B), with viability assays supported by SEM imaging of the ADSC-laden ink after 1-day culture (Figures 5C–F). 2%/3%/0.05% (w/w) Alg/Gel/GO ink showed larger pore size and cell anchoring points than pristine 2%/3% (w/w) Alg/Gel (Figures 5E,F), which is promising for efficient living cell integration and delivery.

### 3.5 3D printing cell containing inks

**FIGURE 6:**
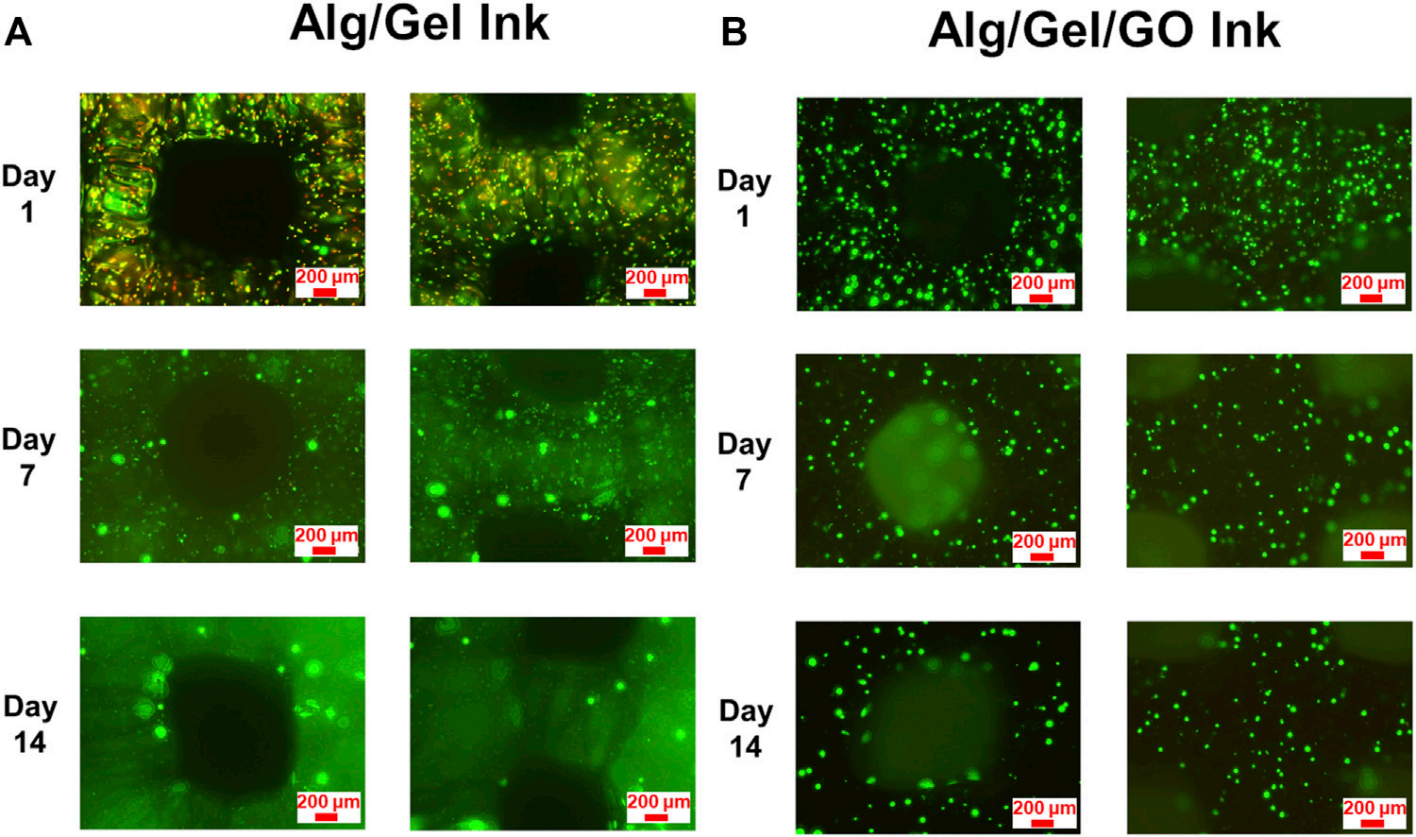
Viability and distribution of 3D printed ADSCs. Fluorescence images of live (Calcein AM; green) and dead (PI; red) cell staining for 3D printed ADSC-laden scaffolds with compositions of **(A)** 2%/3% (w/w) Alg/Gel and **(B)** 2%/3%/0.05% (w/w) Alg/Gel/GO following 14 days culture after printing.

3D printed scaffolds became fragile due to decrosslinking and ensuing degradation during testing. As such, only intact parts were imaged for viability testing (Fannon et al., 2020). Whilst both ADSC-laden 2%/3% (w/w) Alg/Gel and 2%/3%/0.05% (w/w) Alg/Gel/GO bio-inks were 3D printable, the 3D printed GO containing scaffolds remained viable for longer-term culture over 14 days (Supplementary Figures S4, S5), with live cell labelling clearly showing increased viability at days 7 and 14 after printing (Figures 6A,B). In contrast, constructs obtained with a composition of 2%/3% (w/w) Alg/Gel exhibited comparatively higher cell death at day 1 after printing, suggesting GO safeguards cells from sheer stress during printing, and more specifically during extrusion (Figures 6A,B). In addition, GO could effectively adsorb nutritious components on the surface from CM, which may promote cell survival and growth after printing (Zhou et al., 2017). In summary, the higher cell viability for 3D printed GO containing scaffolds indicated cell protection and promotion of proliferation by GO (Figures 6A,B; Supplementary Figure S6). Notwithstanding the differences between scaffolds of both GO and non-GO containing scaffolds, the latter appeared to contain few dead cells by day 14, especially indicative of cell recovery for the pristine scaffolds.

### 3.6 Cell proliferation in 3D printed structures

**FIGURE 7:**
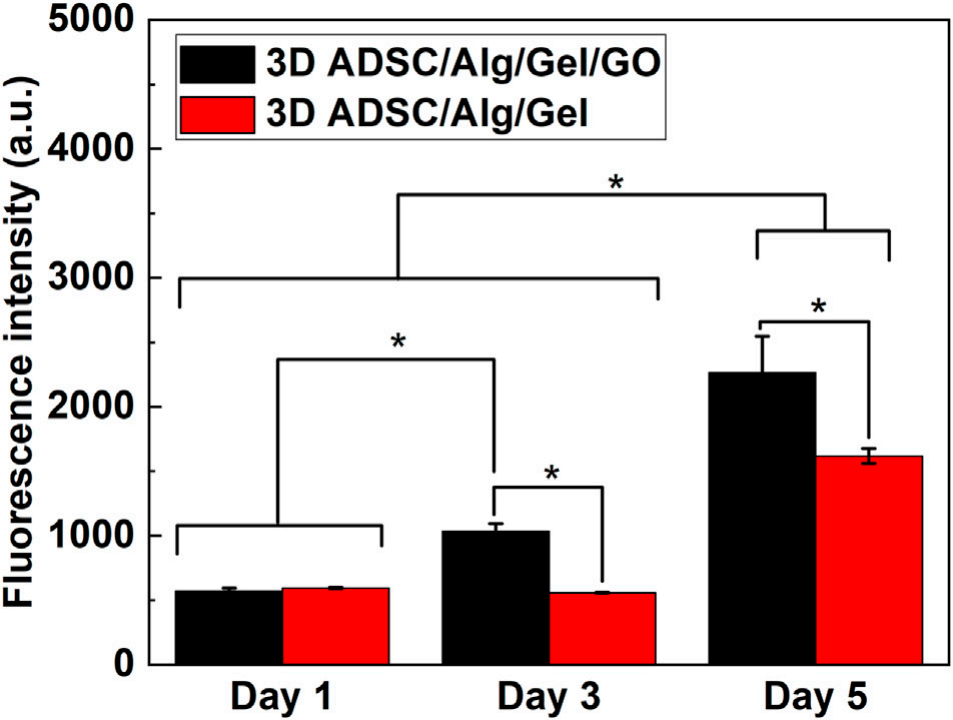
Cell proliferation of 3D printed ADSC-laden scaffolds using compositions of 2%/3% (w/w) Alg/Gel and 2%/3%/0.05% (w/w) Alg/Gel/GO, Mean ± SD, *n* = 3, *p < 0.01.

Analysis of cell proliferation in 3D printed ADSC-laden scaffolds was assessed over 5 days by PrestoBlue assay (Figure 7). Cell proliferation was tested to Day 5, after that scaffolds became fragile afterwards and not amenable for testing with the assay. Statistical analysis revealed that the GO component in the ink has significant effect on promoting ADSC proliferation [F (1, 30) = 82.97, *p* < 0.01], as well as culture time [F (2, 30) = 439.20, *p* < 0.01] and interaction between structure and time [F (2, 30) = 25.02, *p* < 0.01]. Specifically, two-way ANOVA indicated that the number of live cells in 3D printed ADSC-laden scaffolds with a composition of 2%/3%/0.05% (w/w) Alg/Gel/GO was significantly higher compared to ADSC-laden scaffolds with a composition of 2%/3% (w/w) Alg/Gel from day 3 after printing. Cell viability of 3D ADSC-laden scaffolds with a composition of 2%/3%/0.05% (w/w) Alg/Gel/GO was 40% higher than that of 3D ADSC-laden scaffolds with a composition of 2%/3% (w/w) Alg/Gel on day 5. Therefore, ink prepared with GO provided better support for ADSC survival and growth compared to pristine Alg. The influence of GO concentration in the ink and other potential effects on stem cell state and fate will be explored in future studies.

## 4 Conclusion

Here we described the use of GO as a bioactive additive for bio-ink preparation, 3D printing and post-printed cell support. Incorporation of 0.05%–0.1% (w/w) GO in an Alg/Gel hydrogel improves 3D printability of the gel, including printing resolution. Moreover, GO introduced cell-affinity to an otherwise bioinert formulation, likely by providing more bioactive moieties within and on the surface of scaffolds. There were clear benefits of GO addition for ADSCs seeded onto printed scaffolds as well as cells encapsulated through inclusion in the bioink to create ADSC-laden scaffolds. For the latter approach increased cell viability and proliferation immediately after printing and following longer-term culture of printed constructs was determined. Our findings support the inclusion of GO to provide novel bio-ink formulations, with the potential to incorporate other biomaterials, such as collagen, chitosan or cellulose, depending on the tissue engineering challenge at hand.

## Data Availability

The raw data supporting the conclusion of this article will be made available by the authors, without undue reservation.
