# Supplementary material for: Development of 3D printable graphene oxide based bio-ink for cell support and tissue engineering
Source: Front Bioeng Biotechnol. 2022 Oct 18;10:994776. doi: 10.3389/fbioe.2022.994776 (PMC9641498; doi:10.3389/fbioe.2022.994776)
Supplement: Supplementary file 1 [file DataSheet1.docx]

**Development of 3D Printable Graphene Oxide Based Bio-Ink for**

**Cell Support and Tissue Engineering**

*Jianfeng Li^1,2,3*^, Xiao Liu^1^, Jeremy M. Crook^1,4,5,6^, and Gordon G. Wallace^1*^*

^1^ARC Centre of Excellence for Electromaterials Science, Intelligent Polymer Research Institute, AIIM Facility, University of Wollongong, NSW, 2500, Australia

^2^Max Planck Institute of Microstructure Physics, Weinberg 2, Halle (Saale), 06120, Germany

^3^Max Planck-University of Toronto Centre for Neural Science and Technology

^4^Illawarra Health and Medical Research Institute, University of Wollongong, Wollongong, NSW, 2522, Australia

^5^Biomedical Innovation, Chris O’Brien Lifehouse, Camperdown, NSW, 2050, Australia

^6^School of Medical Sciences, Faculty of Medicine and Health, The University of Sydney, Camperdown, NSW 2006, Australia

*Corresponding authors E-mail addresses: [gwallace@uow.edu.au](mailto:gwallace@uow.edu.au) ; jianfeli@mpi-halle.mpg.de


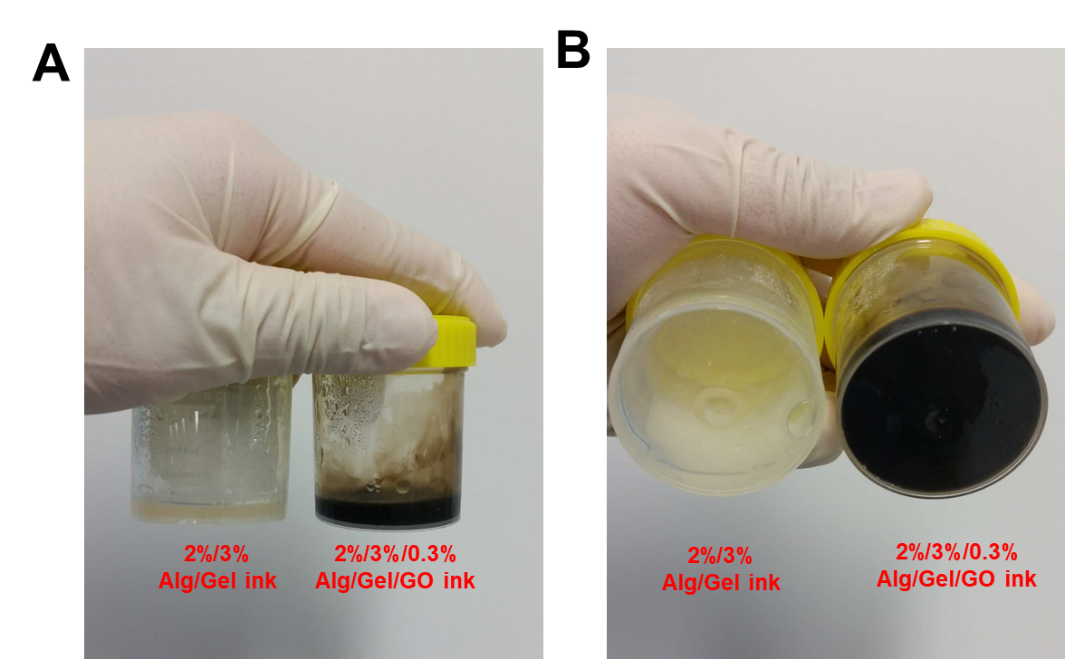


**FIGURE S1. Preparation of pristine 2%/3% (w/w) Alg/Gel ink and 2%/3%/0.3% (w/w) Alg/Gel/GO ink from (A) side view and (B) bottom view.**


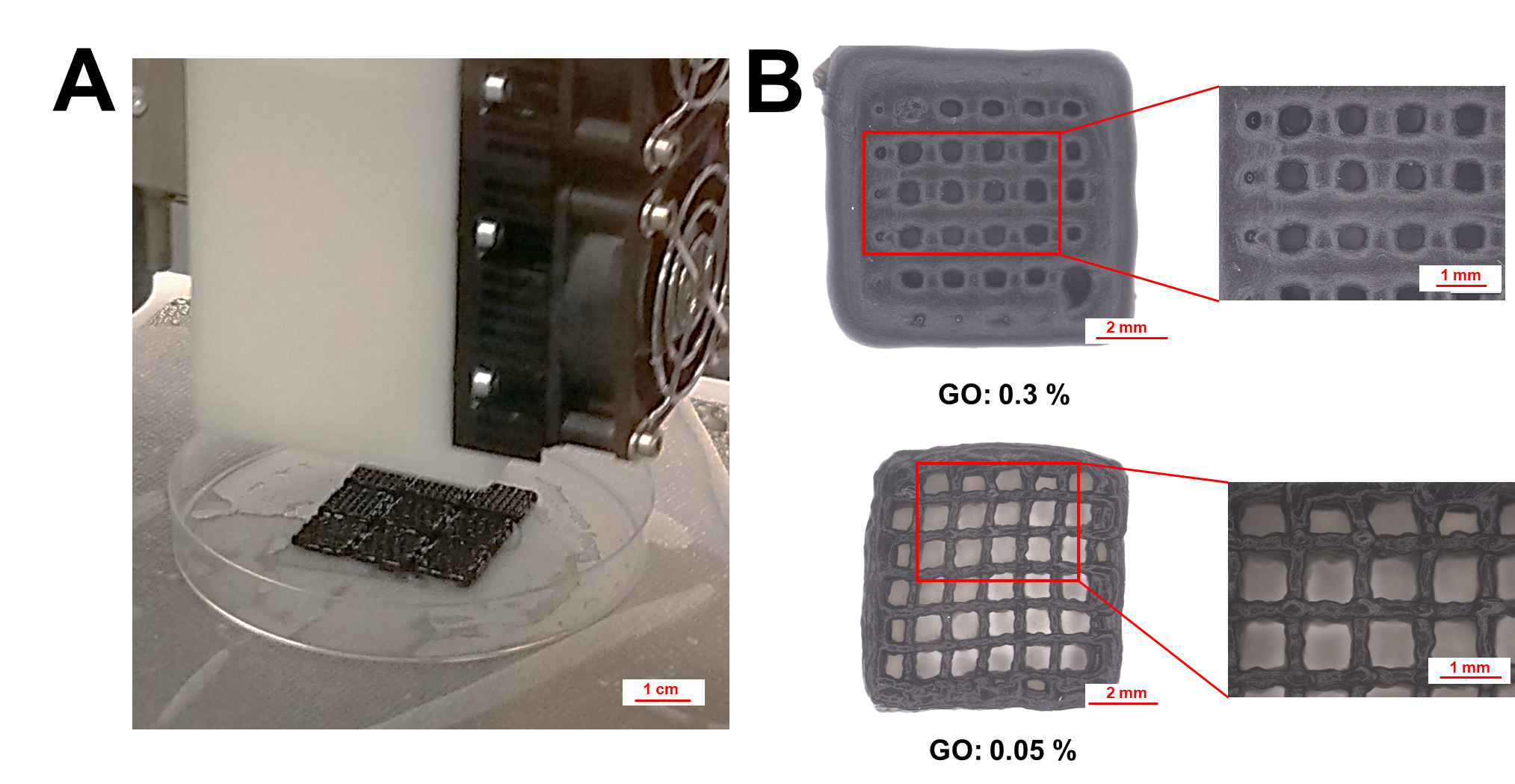


**FIGURE S2. (A) High efficiency 3D printing of GO containing scaffolds in a petri dish on cold stage. (B) 3D printed scaffolds with 0.3% (w/w) and 0.05% (w/w) GO contents, correspondingly.**

**
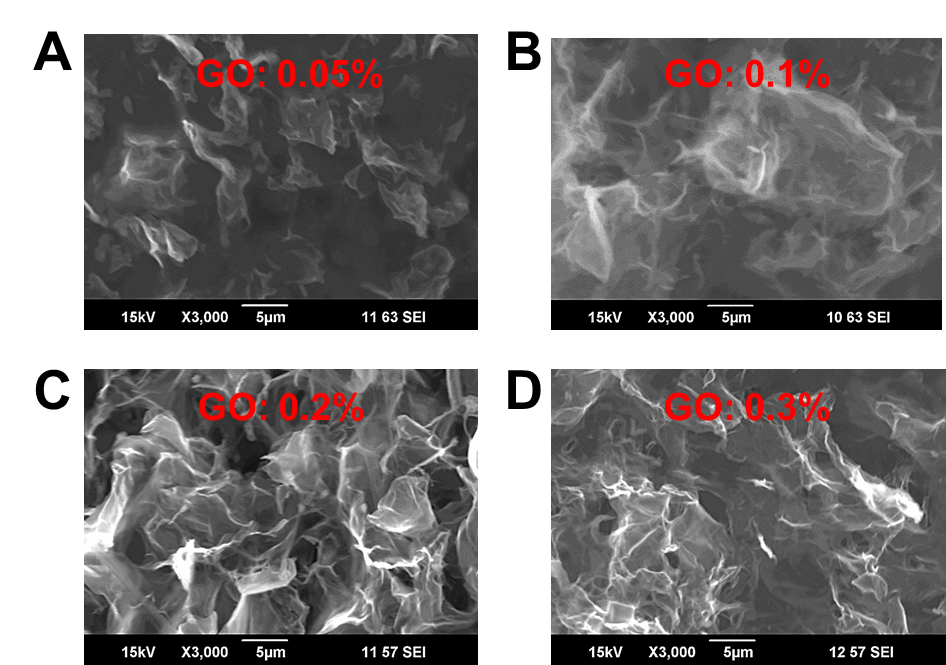
**

**FIGURE S3. Ink morphology with different GO concentrations: (A) 0.05% (w/w), (B) 0.1% (w/w), (C) 0.2% (w/w) and (D) 0.3% (w/w).**


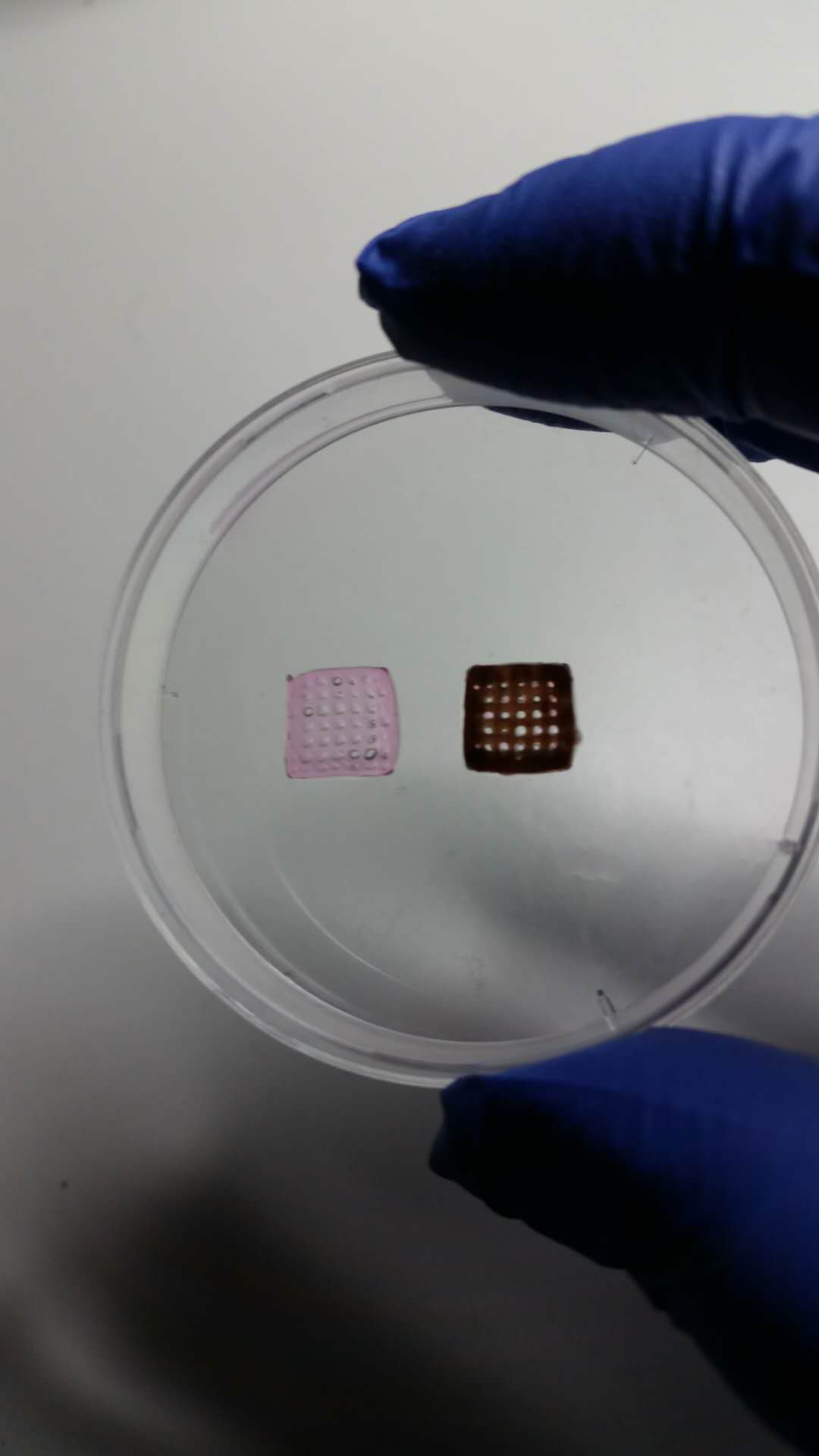


**FIGURE S4. 3D printed ADSC-laden scaffolds with the following compositions of pristine 2%/3% (w/w) Alg/Gel (left) and GO containing 2%/3%/0.05% (w/w) Alg/Gel/GO (right).**


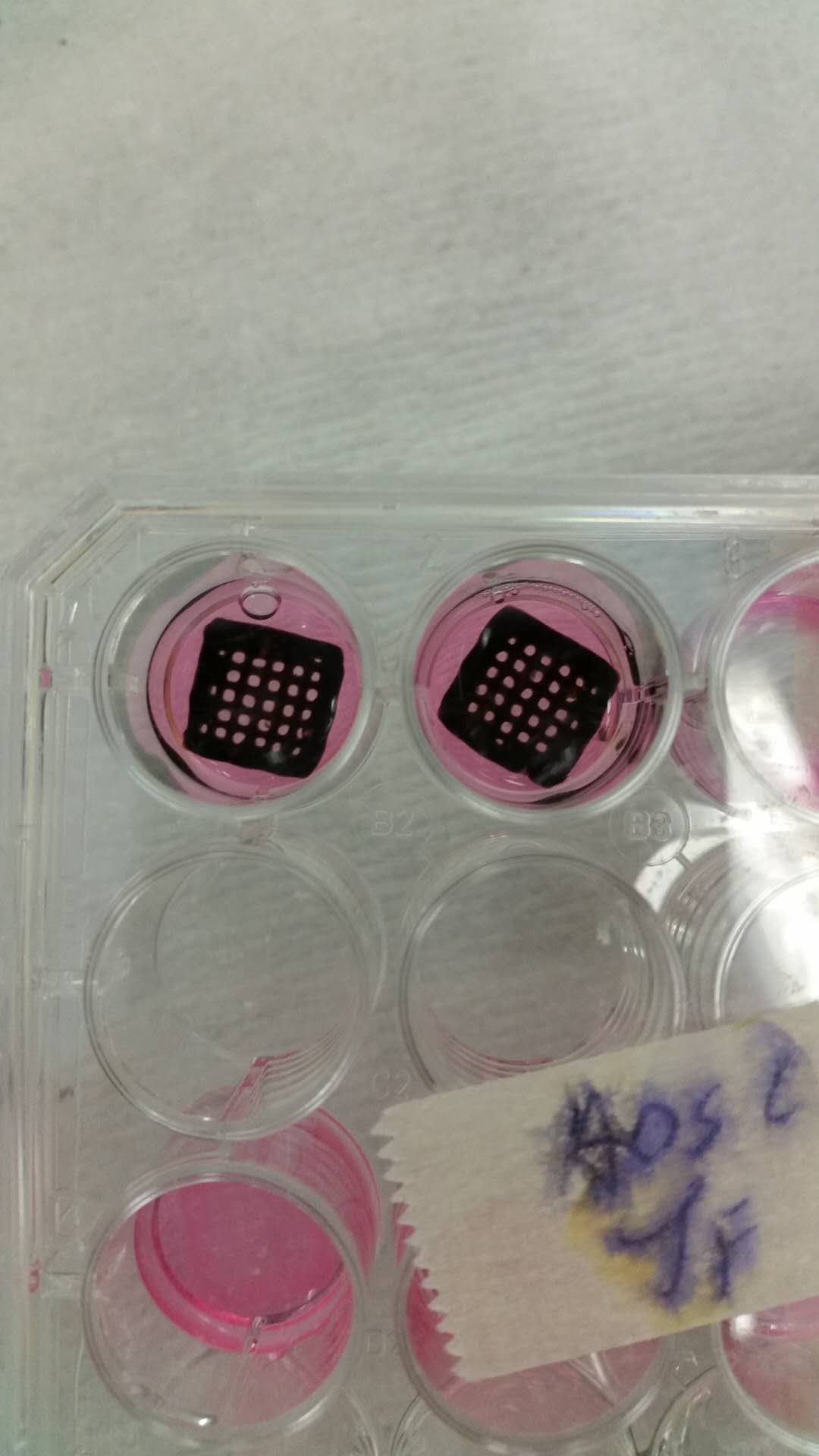


**FIGURE S5. 3D printed ADSC-laden GO containing scaffolds were still integrated after 14 days culture.**


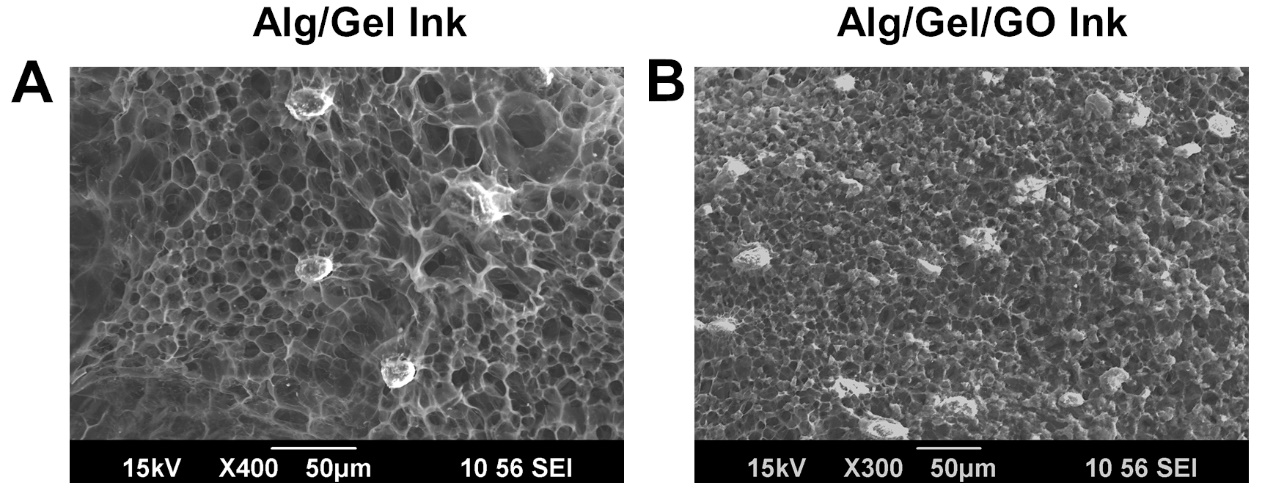


**FIGURE S6. SEM images of ADSC-laden (A) 2%/3% (w/w) Alg/Gel ink and (B) 2%/3%/0.05% (w/w) Alg/Gel/GO ink following 14 days culture after preparation, showing GO promotes cell proliferation.**
